# Endemic type of animal trypanosomiasis is not associated with lower genotype variability of *Trypanosoma congolense* isolates circulating in livestock

**DOI:** 10.1016/j.rvsc.2009.03.003

**Published:** 2009-10

**Authors:** J. Masumu, D. Geysen, P. Van den Bossche

**Affiliations:** a University of Pretoria, Department of Veterinary Tropical Diseases, Onderstepoort, Pretoria, Gauteng 0110, South Africa; b Institute of Tropical Medicine, Animal Health Department, Nationalestraat 155, B-2000 Antwerp, Belgium

**Keywords:** *Trypanosoma congolense*, Savannah type, Endemic area, Variability, AFLP, Zambia

## Abstract

In order to verify whether the low impact on livestock production in endemic areas is related to a low number of trypanosome strains circulating in livestock, 37 *Trypanosoma congolense* isolates collected from cattle in 11 sites in an endemic trypanosomiasis area in Eastern Zambia were characterised for genotype variability using a modified amplified fragment length polymorphism technique (AFLP). Isolates were further cloned to evaluate the occurrence of mixed infections in individuals. The results obtained revealed a high genotype diversity (94.6%) among these isolates. Apart from one site, all isolates gave different AFLP profiles in each of the sites. When clones were compared, three (8%) of the 37 isolates had mixed infections. These results indicate the circulation of a high number of strains in this trypanosomiasis endemic area despite the low impact the disease has on livestock production.

## 1 Introduction

*Trypanosoma congolense* is the main trypanosome species affecting livestock within the tsetse belt areas in most countries in Sub-Saharan Africa. In southern Africa for example, higher prevalence of the disease encountered in cattle is generally attributed to this species i.e. 75% in Mozambique (Sigauque et al., 2000), 95% in Zambia (Simukoko et al., 2007) and 100% in South Africa (Van den Bossche et al., 2006). Even in East (Ohaga et al., 2007; Mugittu et al., 2001) or West (Lefrançois et al., 1998) Africa, *T. congolense* was found to be the most prevalent trypanosome species infecting cattle. However, previous studies have shown that this species is composed of subspecies differing at genetic (Gashumba et al., 1988; Knwoles et al., 1988; Young and Godfrey, 1983) and biological (Bengaly et al., 2002; Reifenberg et al., 1997) level. Furthermore, trypanosome isolates belonging to the same subspecies and circulating in a restricted area have been found to differ in virulence (Masumu et al., 2006a), transmissibility (Masumu et al., 2006b) and antigenicity (Frame et al., 1990; Masake et al., 1987). Such differences were thought to influence the outcome of the disease in different areas. As a consequence, different forms of the disease have been observed in bovine trypanosomiasis with a more acute evolution of the disease, also called epidemic type, where a high disease prevalence and high mortality rate are observed in livestock. This form of the disease was later shown to occur mostly in livestock kept in the vicinity of game areas and thus subjected to infection with trypanosome strains circulating in the sylvatic cycle (Van den Bossche, 2001; Bourn et al., 2001). In other circumstances however, the disease can evolve towards a milder course of the infection called endemic type. This form of the disease has been shown to occur as a consequence of the domestication of trypanosomes in areas where large game animals are rare or absent (Van den Bossche, 2001; Bourn et al., 2001). According to Van den Bossche (2001), the differences in the expression of the disease are influenced by the number of strains where the endemic form characterised by low genotype variability give a more homologous challenge in comparison to the epidemic type of disease. The total number of strains circulating in a trypanosomiasis focus is also an important consideration when considering the application of livestock immunisation using a cocktail of local trypanosome strains.

In Eastern Zambia, *T. congolense* has been found to be the most prevalent trypanosome species infecting cattle (Sinyangwe et al., 2004; Van den Bossche and Rowlands, 2001; Machila et al., 2001). Here also, two different profiles of the disease are found with an endemic form of the disease being observed on the plateau while a more epidemic form is found in the vicinity of the game park in Lwangua valley. Despite the presence of a susceptible cattle breed (e.g. Angoni), the impact of the disease on livestock on the plateau of the Lwangua river where the endemic form occurs was shown to be low (Doran, 2000).

To verify whether this low impact of the disease on livestock production is related to a low number of trypanosome strains circulating in cattle, *T. congolense* isolates were collected and analysed for genotype variability using a modified amplified fragments length polymorphism (AFLP) as has been described by Masumu et al. (2006c).

## 2 Materials and methods

### 2.1 Study area and isolation of trypanosomes

*T. congolense* populations were isolated from communal cattle kept in a trypanosomiasis endemic focus in Eastern Zambia. A total of 37 isolates were collected from 11 sites from Katete and Mambwe districts in a cross-sectional survey conducted in 2003 where 640 cattle were sampled and 11.7% were found to be infected with *T. congolense* using parasitological techniques. All isolates were identified as *T. congolense* Savannah using the PCR-RFLP technique described by Geysen et al. (2003). This area is exempted of large wild animals and *Glossina morsitans morsitans* that is the main vector takes the majority (75%) of blood meals from cattle (Van den Bossche and Staak, 1997). The description of the study area and sampling sites has been reported elsewhere (Masumu et al., 2006a).

### 2.2 Cloning procedure

Trypanosomes of each isolate were multiplied in OF1 mice. When the parasitaemia reached 10^7.8^–10^8.1^ trypanosomes/ml according to Herbert and Lumsden (1976), a drop of blood collected from the tail was diluted in Phosphate buffer Saline Glucose (PSG). The quantity of PSG was than adjusted to reach a concentration of one trypanosome/drop of 5 μl approximately. To ensure that only one trypanosome was used, several drops of this solution were tested using a microscope (400×). Drops that contained more than one trypanosome and those that did not contain any trypanosome were discarded. On the other hand, each drop that contained only one trypanosome was injected into a mouse previously immuno-suppressed using cyclophosphamide (200 mg/kg). An average of 10 mice was used for each isolate. The approval of the ethics consideration was obtained from the Ethics Commission of the Institute of Tropical Medicine, Antwerp, Belgium (Ref DG001-PD-M-TT).

### 2.3 Purification of trypanosomes, DNA extraction and PCR amplifications

To prepare pellet, trypanosomes were multiplied in mice and at the first peak of parasitaemia, infected blood was collected by heart puncture. Trypanosomes were purified using the mini anion exchange column technique (Lanham and Godfrey, 1970). DNA extraction of these pellets was carried out using the phenol-chloroform method. After extraction, DNA concentration was measured by spectrophotometer (Genesys^®^, Spectronic Unicam, USA). PCR amplifications were performed using the modified amplified fragments length polymorphism (AFLP) described by Masumu et al. (2006c). Briefly, 100 ng of DNA template were digested using *Bgl* II enzyme. The digestion and ligation steps were conducted simultaneously for 3 h at 37 °C. PCR amplifications were done using the selective primer A. Gel electrophoresis was carried out on 6% Elchrom gel (polyNAT) and stained using 0.01% SYBR Green I^®^ (Cambrex Bio Science Rockland, USA). Adapters and primers were used as described by Agbo et al. (2002):

Adapters: 5′-CGG ACT AGA GTA CAC TGT C and 3′-C TGA TCT CAT GTG ACA GCT AG

Primers: 5′-GAG TAC ACT GTC GAT CTA.

### 2.4 Genetic analysis

Banding patterns of profiles obtained for each of the isolates were compared. Isolates that presented identical profile were considered as belonging to the same genotype. Clones derived from each of the isolates were compared among themselves as well as with the respective parent isolates. The genotype variability was calculated as the percentage of different profiles or genotypes found among the isolates. In order to check the stability of profiles, comparison of the profiles of 10 clones taken at different peaks during their expansion into mice was done. The genetic profile of each *T. congolense* isolate was analysed using a bionumeric software (Applied Maths, Kortrijk, Belgium). The unweighted pair group method using the average linkage (UPGMA) method was used to generate a dendogram and calculate the percentage of similarities.

## 3 Results

**Fig. 1:**
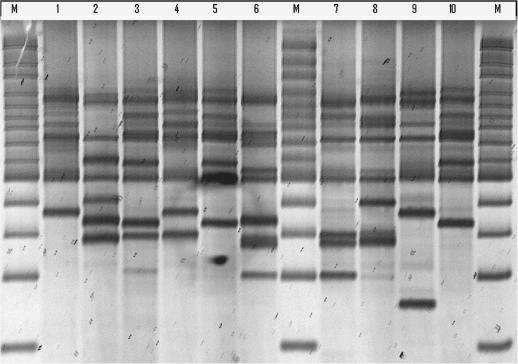
AFLP profiles of 10 *Trypanosoma congolense* isolates collected from an endemic trypanosomiasis area of Eastern Zambia. M: 100+ marker; 1–10: number of isolates.

**Fig. 2:**
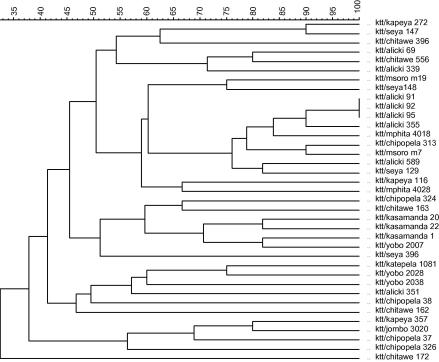
AFLP fingerprint analysis of the 37 *Trypanosoma congolense* Savannah isolates characterised using the selective primer-A. The dendogram was constructed using the unweighted pair group arithmetic mean (UPGMA) method. Percentages of similarity are shown above the dendogram.

All isolates and clones analysed were successfully characterised using the selective primer A. About 10–15 bands ranging from 100 to 3000 bp were generated from various isolates (AFLP profiles of 10 of these isolates are shown in Fig. 1). In total, 35 different genotypes were obtained from the 37 isolates collected, giving 94.6% of genotype variability. Apart from one site (site 1) where three of eight infected cattle were found to be infected with the same genotype, all the other isolates from the different sites gave different profiles suggesting that they belonged to different genotypes. The dendogram generated by UPGMA method of the 37 isolates is shown in Fig. 2. The three isolates that were genetically identical exhibited 100% of similarity. In the remaining isolates the percentage of similarity ranged from 33% to 90%.

A total of 83 clones were obtained from the 37 isolates collected. All clones of 34 isolates had the same genotype profile as the parent isolate showing identical banding patterns. Different genotype profiles were found in clones originated from three isolates giving a prevalence of 8.1% of mixed infections. All results are shown in Table 1. Genotype analysis of clones expanded into different mice and analysed at different peaks of parasitaemia gave identical genotype profiles confirming the reproducibility of the technique (data not shown).

## 4 Discussion

The diversity of pathogenic trypanosomes infecting cattle (e.g. *T. congolense*) in different trypanosomiasis foci is poorly understood. This is mainly due to the lack of a sensitive tool to analyse genotype polymorphism on limited pathogen extracts. In this study, use was made of a modified AFLP that was shown to be sensitive for the characterisation of *T. congolense* isolates (Masumu et al., 2006c). As shown in the dendogram, genotypes characterised using this tool exhibit considerable variations at molecular level. In addition these genetic variations were shown to be associated to considerable differences in various biological characters among these isolates including their pathogenicity in mice (Masumu et al., 2006a), their transmissibility by tsetse flies (Masumu et al., 2006b) as well as their resistance to trypanocides (Delespaux et al., 2008).

In previous studies, high genotype variability was reported using isoenzyme tools in livestock trypanosomes using either *T. vivax* (Dirie et al., 1993; Fasogbon et al., 1990) or *T. congolense* (Gashumba et al., 1988; Young and Godfrey, 1983). In these studies, isolates originated from various hosts and different countries. However, the results of our study suggest that even in a restricted area endemic for trypanosomiasis cattle can be subjected to challenge with several different *T. congolense* strains.

Indeed it was suggested that a low genotype variability would be present in endemic trypanosomiasis areas as a result of elimination of virulent trypanosome strains (Hide et al., 1994). This hypothesis is supported by the fact that virulent trypanosome strains would necessitate frequent treatment or induce higher mortality in susceptible hosts and thus reduce their overall presence. In the area where our isolates were collected, about 20% of the isolates circulating in livestock have been found to exhibit such a virulent profile (Masumu et al., 2006a). However, the majority of isolates had a moderate or low virulence resulting in mild infections. Moreover, further analysis revealed that the presence in livestock of an infection with low virulent trypanosome strains protect animals when challenged with the virulent ones (Masumu et al., in preparation). Under these circumstances, even when infected with virulent trypanosome strains, animals in this area will rarely need treatment as confirmed elsewhere (Van den Bossche et al., 2000). Consequently, the chance for *T. congolense* strains of either high or low virulence to be eliminated through animal death or treatment is low, explaining the maintenance of such a high genotype variability in the study area.

Since, in the field, cattle are continuously challenged by infected tsetse, it is not surprising that a large proportion of cattle harbour infections with multiple trypanosome strains. Similarly the occurrence of mixed infections in *T. brucei* has already been reported (MacLeod et al., 2000, 1999). It should be said that the proportion of mixed infections observed in this study (8% of isolates) could be underestimated. This in particular because of the well known fact that selection will take place due to serial passages in mice before cloning and that cloning was realised during the peak of parasitaemia known to consist of one predominant clone. It is possible that serial cloning during different peaks of parasitaemia or exhaustive cloning could reveal greater mixed infections as previously shown in malaria (Thaithong et al., 1984). However, cloning at different peaks or exhaustive cloning is a laborious procedure when large numbers of isolates need to be examined. Despite this drawback related to the cloning procedure, the presence of mixed infections in this study reflects a situation to be expected as a result of genotype variability and absence of sterile immunity following heterologous challenge in this parasite (Nantulya et al., 1984; Wellde et al., 1981).

As a possible strategy of trypanosomiasis control, local vaccine using a cocktail of trypanosome strains was previously suggested as a result of low serodemes circulating in an endemic area (Masake et al., 1987). However, in another study using trypanosomes circulating in a sylvatic cycle, the use of such local vaccine for the immunisation of livestock kept at the vicinity of the game areas was not found to be practical (Frame et al., 1990). This was supported by the occurrence of a large number of serodemes and subsequent high level of heterologous challenge in *T. congolense* strains circulating in the study area. Since homologous challenge occur only when animals are re-infected with the same trypanosome strain (Nantulya et al., 1984; Wellde et al., 1981), the high genotype variability of *T. congolense* strains in this study suggests that in this trypanosomiasis endemic area and possibly in other areas where the disease displays an endemic profile, livestock can still be subjected to heterologous challenge despite the low impact the disease has on livestock production. Consequently, under these circumstances, vaccination using a cocktail of local *T. congolense* strains does not appear to be a practical option for the immunisation of livestock kept in such domestic cycles as already has been concluded in case of a sylvatic cycle (Frame et al., 1990).

## Figures and Tables

**Table 1:** Number of genotypes, clones and mixed infections of *Trypanosoma congolense* isolates collected from an endemic trypanosomiasis area of Eastern Zambia.

Site number	Number of isolates	Number of genotypes	Number of clones	Number of isolates with mixed infections
1	8	6	21	1
2	5	5	12	–
3	5	5	9	1
4	3	3	7	–
5	3	3	7	–
6	1	1	2	–
7	1	1	2	–
8	2	2	4	–
9	2	2	4	–
10	4	4	8	–
11	3	3	7	1
Total	37	35	83	3
